# Eye and Voice-Controlled Human Machine Interface System for Wheelchairs Using Image Gradient Approach

**DOI:** 10.3390/s20195510

**Published:** 2020-09-26

**Authors:** Saba Anwer, Asim Waris, Hajrah Sultan, Shahid Ikramullah Butt, Muhammad Hamza Zafar, Moaz Sarwar, Imran Khan Niazi, Muhammad Shafique, Amit N. Pujari

**Affiliations:** 1 School of Mechanical and Manufacturing Engineering, National University of Sciences and Technology, Islamabad 45200, Pakistan; sanwer.bmes19smme@student.nust.edu.pk (S.A.); hsultan.bmes19smme@student.nust.edu.pk (H.S.); drshahid@smme.nust.edu.pk (S.I.B.); 2 Department of Electrical Engineering, University of Engineering and Technology Lahore-FSD Campus, Faisalabad 38000, Pakistan; Hamzazafar214@gmail.com; 3 Department of Computer Sciences, Government College University, Faisalabad 38000, Pakistan; moazejaaz@gmail.com; 4 Center of Chiropractic Research, New Zealand College of Chiropractic, Auckland 0600, New Zealand; imran.niazi@nzchiro.co.nz; 5 Department of Health Science and Technology, Center for Sensory-Motor Interaction, Aalborg University, 9000 Alborg, Denmark; 6 Faculty of Health and Environmental Sciences, Health and Rehabilitation Research Institute, AUT University, Auckland 0627, New Zealand; 7 Head of Department, Biomedical Engineering, Riphah International University, Islamabad 45710, Pakistan; muhammad.shafique@riphah.edu.pk; 8 School of Engineering and Technology, University of Hertfordshire, Hatfield AL10 9AB, UK; amit.pujari@ieee.org; 9 School of Engineering, University of Aberdeen, Aberdeen AB24 3FX, UK

**Keywords:** human machine interface (HMI), rehabilitation, wheelchair, quadriplegia, Raspberry Pi, image gradient, AMR voice, Open-CV, image processing

## Abstract

Rehabilitative mobility aids are being used extensively for physically impaired people. Efforts are being made to develop human machine interfaces (HMIs), manipulating the biosignals to better control the electromechanical mobility aids, especially the wheelchairs. Creating precise control commands such as move forward, left, right, backward and stop, via biosignals, in an appropriate HMI is the actual challenge, as the people with a high level of disability (quadriplegia and paralysis, etc.) are unable to drive conventional wheelchairs. Therefore, a novel system driven by optical signals addressing the needs of such a physically impaired population is introduced in this paper. The present system is divided into two parts: the first part comprises of detection of eyeball movements together with the processing of the optical signal, and the second part encompasses the mechanical assembly module, i.e., control of the wheelchair through motor driving circuitry. A web camera is used to capture real-time images. The processor used is Raspberry-Pi with Linux operating system. In order to make the system more congenial and reliable, the voice-controlled mode is incorporated in the wheelchair. To appraise the system’s performance, a basic wheelchair skill test (WST) is carried out. Basic skills like movement on plain and rough surfaces in forward, reverse direction and turning capability were analyzed for easier comparison with other existing wheelchair setups on the bases of controlling mechanisms, compatibility, design models, and usability in diverse conditions. System successfully operates with average response time of 3 s for eye and 3.4 s for voice control mode.

## 1. Introduction

Significant strides made in the fields of rehabilitation, artificial intelligence (AI) (especially around the implementation of complex algorithms for analysis and interpretation of human cognition), and human machine interfaces (HMIs), have opened a new evolutionary pathway for the development of smart mobility aids [1]. People who accidentally lose their lower limbs or suffer from conditions such as quadriplegia or stroke, resulting in paralysis, and muscle stiffness are unable to make use of conventional wheelchairs [2]. Researchers across the world are engaged in developing medical devices/rehabilitation aids for physically challenged populations, such as quadriplegics, to enable them to carry out their daily work without or with minimal assistance from caregivers and nurses, etc. [3]. They are thus increasing the self-esteem and functional capabilities of such patients with the ultimate goal of improving the patients’ quality of life. Biosignals recorded through electroencephalography (EEG), electromyography (EMG), and electrooculography (EOG), etc. [4,5], have been exploited by researchers in developing smart, responsive and real-time rehabilitative control systems.

More recently, eye gesture control-based systems have gained significant attention due to the fact that even in the most seriously physically challenged population, such as quadriplegics, eye movements are still intact; the main operating mechanism of the eye-controlled based systems [6]. Therefore, keeping in view this fact and the need to advance the adoption of independent mobile rehabilitation technology, such as smart wheelchairs and walkers, a distinctive eyeball movement-based technique for controlling a wheelchair is presented, leading to increased patient comfort.

The main purpose is to design an autonomous system that requires minimal manual assistance and thereby provides wheelchair users with a sense of confidence, competence, and independence. As such, the presented system should be easy to use for a paralyzed individual with a severe lower limb disability. Additionally, the system also incorporated voice-controlled technology [7]. The system is low-cost, easily manageable, scalable, and designed with the user’s comfort in mind.

Algorithms currently used for face detection and feature extraction in eye controlled systems include Hough circle (feature extraction based approach used to detect circular objects in image processing) [8] and active infrared illumination (uses IR sensors to detect the eye movement and emit IR radiation ranging from 700–1000 nm in the electromagnetic spectrum) [9]. Although these methods accurately localize eyeball position and are relatively simple compared to other existing techniques, e.g., Haar cascade [10], they do have some drawbacks. For example, the Hough circle technique (CHT), when applied to discrete images, demands a large storage capacity, as well as computing power [11] and an active infrared illumination technique, which can cause irreversible damage to eyes, resulting in the loss of efficiency of a working body organ, thus worsening the situation [12]. Moreover, these algorithms sometimes fail during complex situations, for example, the low resolution of images and low contrast conditions.

Although the use of biosignals, i.e., EEG (electroencephalogram) and EMG (electromyography) is widely accepted to develop HMIs, for example, EMG based physiotherapy devices [13] and EEG based diagnostic medical equipment [14], however, these systems come with wearable technology which is not practical or comfortable in the case of mobility assistive mechanization. In EEG and EMG based systems, a user also must be in contact with electrodes while using the device/wheelchair, thus making the system cumbersome and uncomfortable. Further, electrodes (for EEG, EMG) are susceptible to a range of issues. For example, signals may be contaminated by a variety of noises at the electrode–skin interface, which can lead to contamination of the acquired biosignals [15,16]. The electrode issues include (1) motion artifacts, which occur when a force impulse travels through muscle causing an unwanted movement at the skin–electrode interface; (2) inherent noise in electrical components, which cannot be completely removed; (3) ambient noise occurring due to electromagnetic radiation as the human body is persistently exposed to this radiation; and (4) power line interference and other disruptions like baseline shifts (due to the excessive motion of cable, baseline shows a significant shift from the actual position). All these noises attenuate the desired signal causing undesired results [17].

A variety of control techniques have been used for creating assistive HMIs. However, all these systems had their own limitations in terms of operating efficiency. For example, head motion controlled and hand gesture operated wheelchairs [18,19], both of these controlling modes (head and hand) include the use of flex sensor and accelerometer. Sometimes a sensor’s efficiency is greatly affected by environmental factors (temperature, dust and humidity). Once contaminated, this can cause controllability issues. Moreover, an accelerometer has a fixed operating range limiting its application, thus obstructing the way actual acceleration is read.

Although distinct eye motion-controlled wheelchairs were developed claiming to assist disabled individuals, they are limited in their functioning capability and comfort level; as these systems eyeball movements are processed using software, such as MATLAB, and computing devices (laptop, etc.) are required to be carried all the time, which occupy substantial space, making the system cumbersome and expensive [20,21].

Considering the limitations of existing systems discussed above, a system that tries to overcome these limitations and ensures patient safety and comfort, as well as be scalable and highly functional, is presented here. The operating system is installed in Raspberry Pi, and the language used for processing the eyeball movements in real-time captured images is C++, using the Open computer vision (Open CV) library [22]. As the introduced system is compact in design, it is relatively easy to install in a wheelchair. Along with the eyeball control option, this system can also be controlled via voice commands, increasing the system’s adaptability and usability.

## 2. System Model

**Figure 1:**
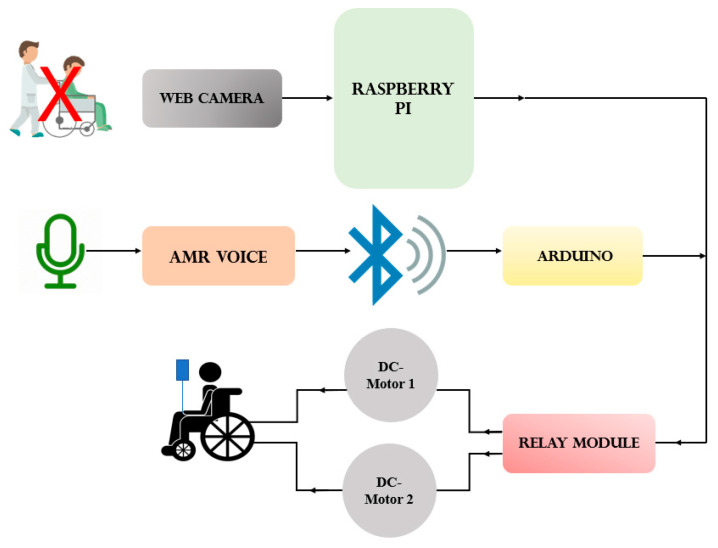
Block diagram of the system.

Components of the system model and their interaction with each other are shown as a block diagram (Figure 1). The webcam is fixed to a vertical pole precisely in front of the user’s eye, and this webcam is connected to Raspberry Pi so that it can continuously capture images of the user’s eyeball movement and respond. Raspberry Pi installed with Open CV has an image processing capability and generates an actuation signal. Raspberry Pi is coupled with the motor driving circuitry, which is responsible for directing the wheelchair according to a given command.

For the voice control mode, an audio signal is fed to the Arduino via a Bluetooth module (HC-05 BT). Arduino is programmed to process these voice commands and generate the required drive signal. Arduino is wired with a switching circuit (four-channel relay module) responsible for driving the motors in the designated direction.

**Figure 2:**
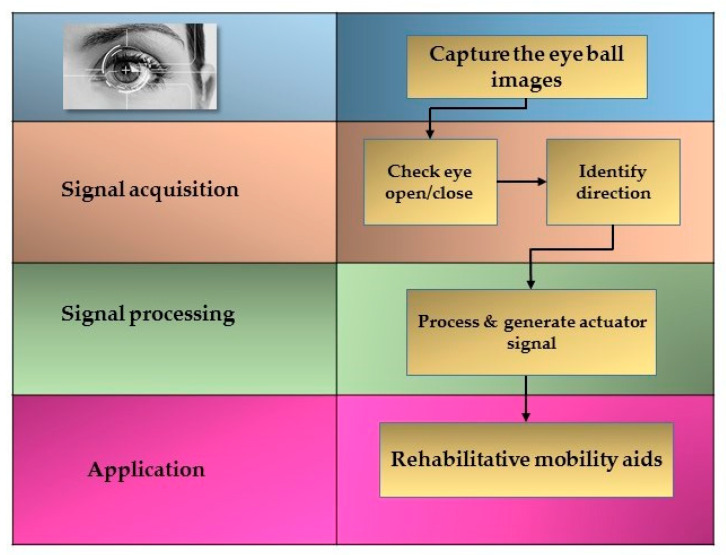
General process flow block diagram.

Figure 1 depicts the complete mechanical control mechanism of the system, including all the major components, i.e., Raspberry Pi, webcam, power supply, microphone, Arduino, DC-motors, motor driving circuitry, and Bluetooth device. The system works using real-time data acquisition, with Raspberry Pi as the main controller used for eyeball tracking. Raspberry Pi is a low-cost single-board embedded processor, which thus reduces the complexity of the system and is suitable for real-time applications. In the present system, a distance of approximately 1–1.5 feet is maintained between the user’s eye and the webcam. To keep the costs down, and for recognition accuracy and processing speed, a 1080P webcam is used. Figure 2 describes the process flow of the eye control mechanism. First, the webcam captures real-time images of the eyeball and then identifies whether the eye is open or closed. If closed, then images are recaptured and analyzed again to identify the direction of the eyeball. Once the eyeball direction is confirmed, the signal is processed, and an actuator signal is generated, which is then fed to the motor driving circuitry of the wheelchair.

## 3. Methodology

**Figure 3:**
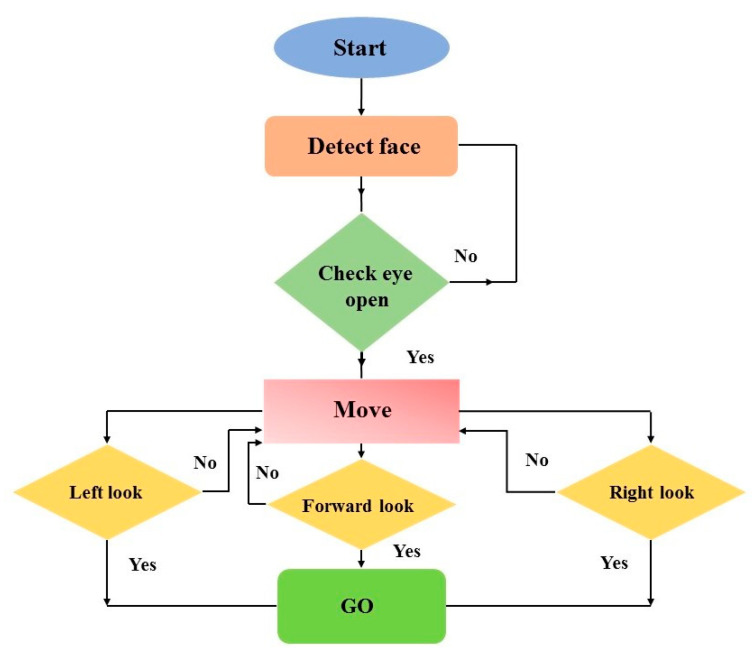
Functional flow chart of eye control system.

Figure 3 illustrates the complete functional flow chart of the eye control system. The system begins with capturing images through the webcam. After capturing a real-time image, the system detects the face and then extracts eye images.

A complete flow chart is defined using state, condition, and decision boxes. State boxes are denoted by rectangular shapes with round corners, decision boxes are diamond-shaped, and condition boxes are rectangular with sharp corners. State boxes represent the status of the system (i.e., moving or not), decision boxes describe direction (i.e., left, right or forward), and condition boxes give information about the system’s working condition (i.e., face detection or driving the wheelchair).

Paths indicate the process flow. For example, after initialization, the system detects the face, and then it checks whether the eye is open or closed, as shown in Figure 3. After this, the eye pupil’s position is identified, i.e., whether the user is looking forward, left, or right. After the eyeball position is identified, this image is processed. Raspberry Pi then generates an actuator signal, which is fed to a switching circuit (relay) to drive the motors accordingly.

## 4. Algorithm

The most challenging task is to locate the eyeball movement. This task is accomplished using the image gradient approach described below. As mentioned earlier, various other techniques have been used to locate eyeballs. Although they provide accurate results and are used in commercially available eye-tracking and face recognition systems, they are not easy or practical to use as they come with head-mounted or wearable technology.

In the present system, a feature-based approach is applied that can accurately locate the eye centers using a webcam, even in low-resolution videos and images [23]. A simple and easy approach is applied, which defines the center of circular objects as the location where the intersection of multiple image gradients occurs.

### Eye Center Localization by Gradient Vectors

By considering a vector field comprising of image gradients, geometrically, the eyeball center can be located. A fast iterative scheme is achieved by using a mathematical formula [23]. The formula describes a relation between the conceivable center and all the image gradients directed towards it.

**Figure 4:**
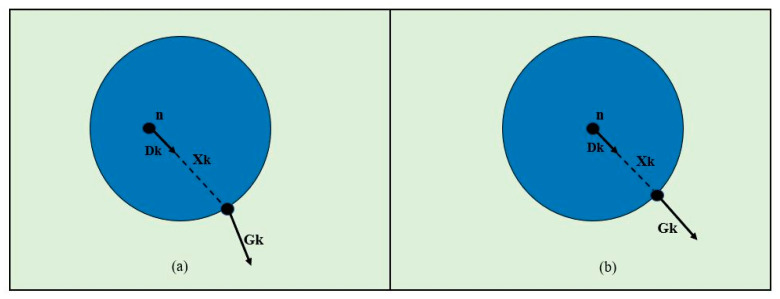
A contrived example is having a dark-colored circle against a light background, similar to an iris and sclera. In (**a**) *D_k_* (displacement vector) and *G_k_* (gradient vector) do not have same direction but in (**b**) the orientations are same.

Suppose n is a possible center, and *G_k_* is the gradient vector. If the position of this gradient vector *G_k_* is *X_k_* then the direction of displacement vector *D_k_* should be the same as gradient vector *G_k_* (Figure 4).

If the vector field of image gradients is used, then this vector field can be exploited by calculating the dot products between displacement *D_k_* and gradient vectors *G_k_,* by using the (1). Center n of a circular object in an image with pixel positions *X_k,_* (where *k* ∈ {1, ……, N}) is measured by (1).

(1) n=1N∑i=1N(Dk^T·Gk)2

(2) Dk=xk−n||xk−n||^2

∀ *k*: || *Gk* ||*2* = *1* (3)

To get an equal weight for all the pixel positions, displacement vectors *(D_k_)* are scaled to unit length (2). Robustness to the linear variations in luminous conditions can also be improved by scaling gradient vectors *G_k_* to unit length (3).

Calculations can be simplified by considering only the gradient vectors *(G_K_)*. Partial derivatives are computed to get the image gradients (4).

(4) $$G_{k}=\frac{\delta_{P}\left(x_{k},y_{k}\right)}{\delta X_{k}},\frac{\delta_{P}\left(x_{k},y_{k}\right)}{\delta Y_{k}}$$

**Figure 5:**
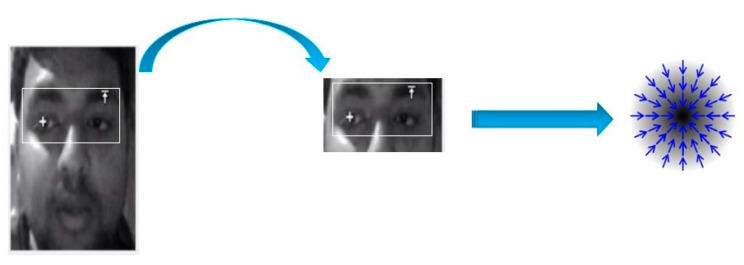
Eye core located (**left**) indicated with white mark in the presented system, using image gradients symbolically directed towards the center (**Right**).

When gradients are computed, it is possible that images have extra structures, i.e., hairs, spectacles, and eyebrows. These structures are responsible for gradients that do not possess the same direction as image gradients of the eye. Due to these structures, eye center computation may become difficult. To overcome this difficulty, the threshold is applied to the objective function. This threshold is based on the maximum value, which eliminates all other remaining entities associated with the desired image boundary. After this, a maximum of prevailing entities is computed, and its position is taken as the eye center (Figure 5). This threshold does not have any negative impact on estimating the eye center. In the present system, the threshold is taken as 85% (0.85) of the overall maximum.

## 5. Eye Control Mode

During eye control mode, webcam captures the real-time images and sends them to Raspberry Pi, Raspberry Pi processes them and generates an actuator signal. Raspberry Pi is further wired with a 4-channel relay module (switching circuit) responsible for driving the two DC-motors, thus making an efficient, reliable, and easily functional system.

In the present system, Raspberry Pi model 3B is used as the main controller. Its working resembles a CPU. It has its own RAM, ROM, internet port, 36 connection pins, four supply pins, four USB (universal serial bus) ports and one memory card holder up to 32 GB [24]. Raspberry Pi is Raspbian supportive hardware. Raspbian runs on the Linux operating system. Processing to track eye is done in Open CV 3.0 [17] (open computer vision) library. This library is commonly used for image processing. Open CV is installed under a BSD license, which is free for both commercial and academic purposes. Open CV has C++, C, and Java interfaces and is the most suitable platform for real-time applications.

**Figure 6:**
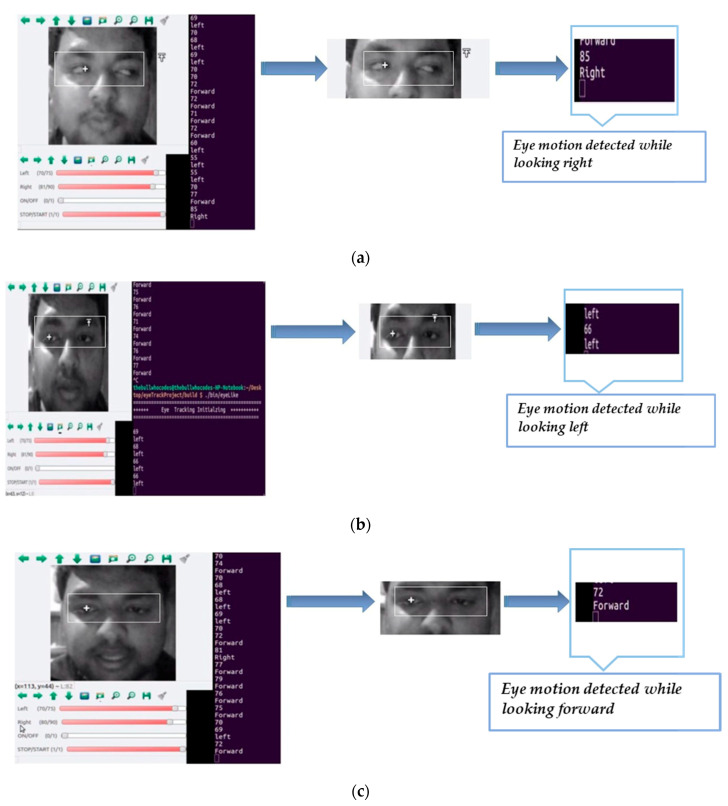
Designed system’s results for eye control mode (**a**) right, (**b**) left, and (**c**) forward.

Integrating Open CV, Linux operating system, and C++ language with hardware has improved the present system’s constancy compared to other existing systems (mentioned earlier) and has reduced processing latency. These features have also improved the system’s compatibility and level of convenience for the user. Figure 6 shows the results of detecting the eye center for right, left and forward commands, respectively. Through turning the wheelchair and then looking forward will move it in reverse direction safely as this system has been designed for an extreme disability (quadriplegic patient).

## 6. Vocal Control Mode

**Figure 7:**
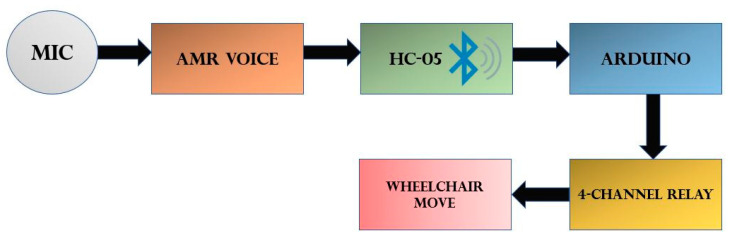
Block diagram for voice control mode.

For voice control mode on the wheelchair, the adaptive multi-rate AMR voice app is used with four commands ON (for forwarding), left, right and stop. Arduino UNO is used as the main controller. Other main components of voice control mode are microphone, HC-05 Bluetooth module, and AMR voice recognition. The block diagram in Figure 7 depicts the functionality flow between all components of voice control mode.

For voice control, the microphone is connected with Arduino via the HC-05 Bluetooth module. Arduino transfers actuator signal to motor driving circuitry, which is a four-channel relay module. Two DC-motors are powered by two chargeable 12-V dry batteries (Figure 7).

## 7. Mechanical Assembly

The most important aspect during the mechanical assembly of the wheelchair is the proper selection of frames. This is necessary for the successful installation of all essential components in the wheelchair. During this phase, the patient’s comfort and compatibility are the top priorities. Moreover, a wheelchair’s material must be resistant to corrosion for its long-lasting utilization, and its posture should be such that the patient’s weight is evenly distributed to avoid pain and pressure sores [25].

**Figure 8:**
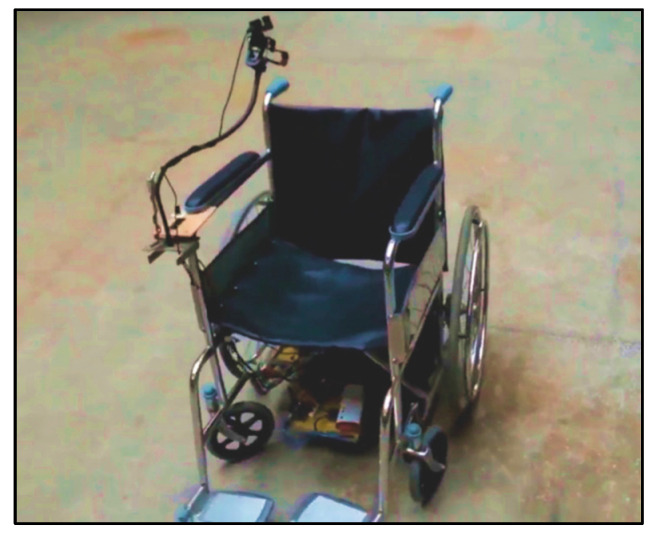
Wheelchair model with all essential components installed appropriately.

**Figure 9:**
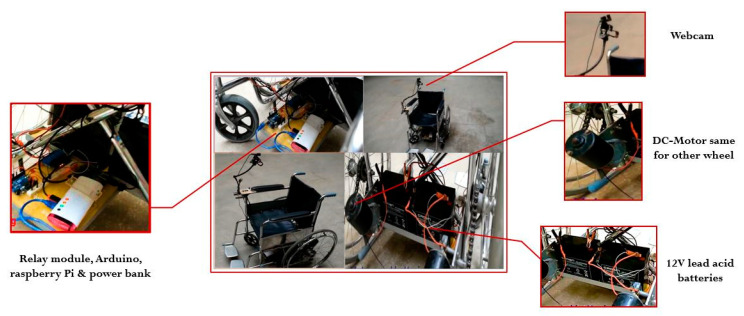
Complete mechanical assembly of wheelchair.

In the present wheelchair design (Figure 8 and Figure 9), all the required components (batteries, motors, controllers and relays) are placed appropriately on a designated platform (length = 12 inches, width = 9 inches, thickness = 5 inches) welded to the wheelchair underneath the seat. A low weight frame is used so that a wheelchair can easily be propelled. Total weight of wheelchair is 17.35 kg (frame = 14.5 kg, other components = 2.85 kg). Further, the camera is located in such a position so that the user can easily gaze into the camera while remaining in the comfort zone, thus avoiding any tiredness.

Two 12-V permanent magnet DC-motors are used in this system with 95 W output power. Further, the system is incorporated with two 12-V, rechargeable, lead-acid batteries for its appropriate functioning. The gear ratio used was 1:20.

## 8. Basic Skills Performance Test for Wheelchair

Wheelchair technology is diverse in nature. Therefore, a basic wheelchair skill test (WST) was carried out to analyze the system’s efficiency and response time. This testing approach has advantages of being easy to manage, requires minimum testing equipment, is inexpensive, and has adequate measurement properties to quantify the performance of wheelchair movements. Results of WST can provide crucial data about the test subject’s performance. For example, whether the subjects were able to accomplish the assigned movement task successfully up to the marked distance and the corresponding response time of the system. Thus, the results of WST are representative of the range of movement of the wheelchair that may be required to be performed regularly by the disabled.

The most suitable term used for an individual selected as the object of testing is ‘subject’ because he/she may be a researcher, caretaker, user, or health care student. However, it is necessary that the test subject meets the same criteria as specified for the wheelchair user. For example, he/she must remain within the designated space and operate a wheelchair as will be operated by a disabled individual.

Therefore, in order to assess the mobility and working proficiency of the wheelchair, a simple test was conducted. The main objective was to ensure and record a chair’s maneuverability, performance, and user compatibility. Basic skills for which the presented setup of the wheelchair was tested included (Table 1).

## 9. Results

**Figure 10:**
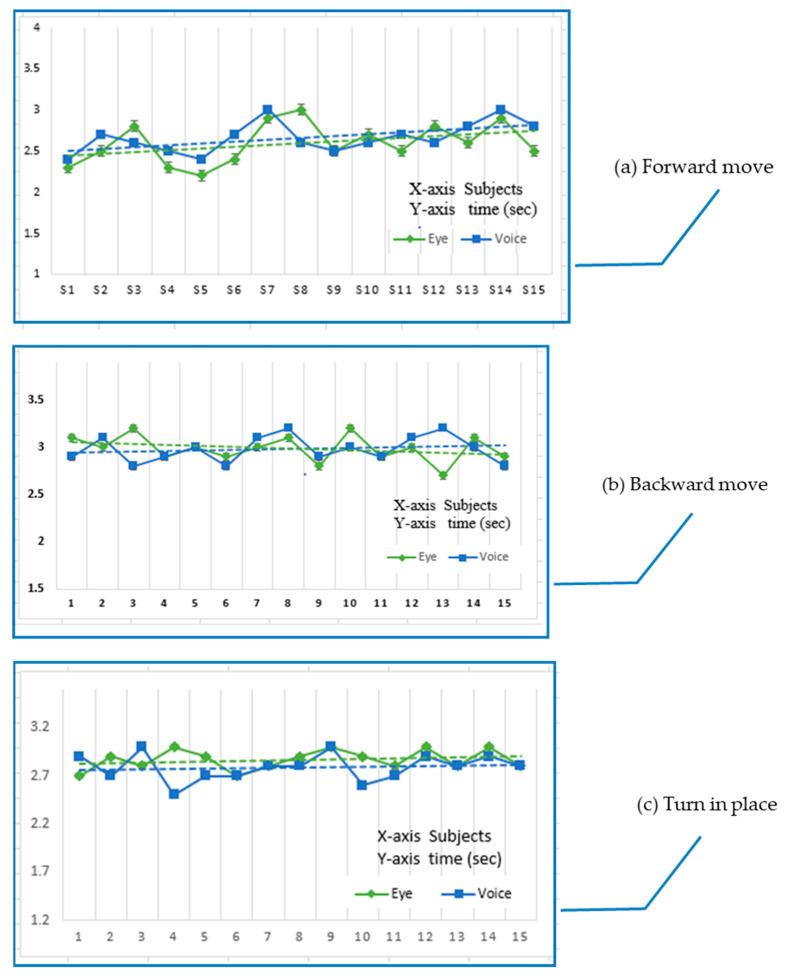

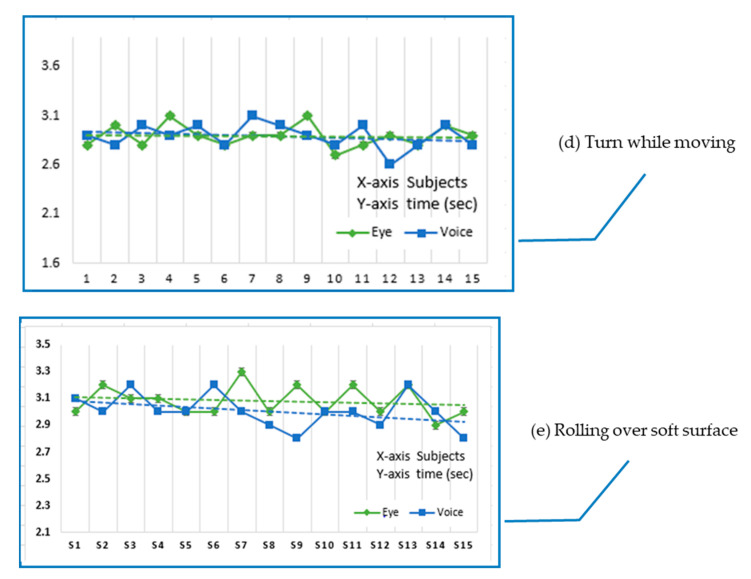
Graphical representation of a basic wheelchair skill test (WST). (**a**) Forward move on a plain surface. (**b**) Backward move over the plain surface. (**c**) Turn in place. (**d**) Turn while moving (**e**) Rolling over a soft surface.

The data obtained from a basic WST-wheelchair skill test are represented graphically in (Figure 10). The presented system was tested with 15 test subjects, in an age range of 20–30 years. All the testing subjects were healthy and were asked to drive the wheelchair via eye and voice control modes separately through a fixed distance of 25 m. All participants operated the wheelchair up to the targeted distance separately for each movement (forward, backward, rollover soft surface, turn left and right). The main goal was to note the system’s response time for both optical and vocal commands, separately.

Moreover, the system’s other attributes, including mobility, rear and caster wheel motion, and compatibility with the user, were also analyzed. Wheelchair processing times were noted with a stopwatch and later compared graphically. The mean response times for each skill were computed.

All the test subjects successfully covered the 25-m distance without any intervention of spotter/researcher. The system was positively responsive, with 99% of participants without any false-positive results.

Table 1 shows the system’s mean response time for each basic skill from 15 participants. It is important to note that the participants had an age range of 20–30 years, disparate eye colors, weights, and vocal pitches. The system was tested separately for both (eye and voice) operating modes, and there was no significant difference between their operating proficiencies—the mean response time was calculated for each movement for each mode (Table 2).

## 10. Discussions

The aim was to develop a wireless system to assist patients with significant disabilities (stroke & quadriplegia). Thus, an eye and voice-controlled wheelchair system, which overcomes almost all the issues encountered in previous HMIs, is presented here. The present system removes the need to carry a personal computer or any wearable electrode band like EMG, hand and head gesture-controlled systems [4,5,6,17]. Furthermore, there are no harmful side effects on human eyes, as experience due to infrared accumulation system, which causes gradual but irreparable damage to the eyes [13]. Thus, the presented system that is safer, quickly responsive, compact, convenient, and user-friendly as compared to other existing wheelchair setups.

Moreover, a small basic WST-wheelchair skill test was also carried out to demonstrate the presented system’s working capability and quantify its response times. The presented system successfully responds between 2.5–3.0 s on the plain surface; however, while moving on soft surface response time was slightly higher as it is relatively difficult to propel the wheelchair on such surfaces due to their high resistance and bumpy appearance (grass, dirt, carpet and dirt). Weight-relief is an important property that is necessary to be considered while designing any rehabilitation device. If such factors are ignored, it may take any body part under pressure causing pressure sores. In the present system, this aspect was carefully considered while integrating the components. The camera was adjusted such that the user does not need to put any extra effort into looking into the camera. Users can easily drive the wheelchair via eye or voice command while remaining in a comfortable position, thus avoiding potential tiredness. Overall, the designed system is proficient, feasible, comfortable, and safe to use. However, the system is not without its limitations. Although the image processing technique used has a relative superiority in processing, these techniques sometimes malfunction in the dark due to variation in illumination. In the existing setup, a 12-V LED is incorporated to compensate for this problem to some extent; however, in the near future, FPGA-field programmable gate array systems may be used to improve the processing speed and make the system more synchronized with environmental variations and user needs.

## 11. Conclusions

An eye and voice-controlled interface for a wheelchair to assist the mobility of physically impaired people has been designed so that they may be able to perform their daily life activities without additional support from a caregiver or healthcare professional.

## Figures and Tables

**Table 1:** Basic skills, the designed wheel chair is capable of.

Basic Attributes
Relief from pressure
Forward move
Backward move
Rollover soft surface
Stop
Turn while moving
Turn in place

**Table 2:** System response for WST.

Attribute	Mean Response Time
Forward move on a plain surface	Eye = 2.53 s, voice =2.8 s
Reverse move over a plain surface	Eye = 3.4 s, voice = 3.0 s
Turn in place	Eye = 2.86 s, voice = 2.9 s
Turn while moving	Eye = 2.78 s, voice = 2.89 s
Rolling over a soft surface (grass)	Eye = 3.46 s, voice = 3.5 s
